# Empowering leadership and job satisfaction of academic staff in Palestinian universities: Implications of leader-member exchange and trust in leader

**DOI:** 10.3389/fpsyg.2022.1065545

**Published:** 2022-12-21

**Authors:** Ibrahim Horoub, Pouya Zargar

**Affiliations:** ^1^Department of Communication, Girne American University, Kyrenia, Cyprus; ^2^Department of Business, Girne American University, Kyrenia, Cyprus

**Keywords:** empowering leadership, trust in leader, university teachers, job satisfaction, leader-member exchange, educational psychology

## Abstract

**Introduction:**

In the aftermath of global pandemic of COVID-19, many sectors faced severe challenges to maintain security, health (psychological, and physical), and steer through the crisis by sup-porting the society.

**Methods:**

Through a quantitative approach a total of 250 surveys were distributed after a pilot test. Specifically, this research gathers data from 178 (71.2% response rate) university teachers from different universities across Palestine via surveys that address the role of empowering leaders on job satisfaction among teachers. The proposed model of the re-search was analyzed using Smart-PLS and PLS-SEM technique.

**Discussion and Results:**

The academic sector was disrupted and faced extreme changes during the pandemic, rendering teachers vulnerable and thus, role of leaders more crucial. Building on job demand-resources model, and social exchange theory, the current study examines the moderating effect of leader-member exchange (LMX) for increasing job satisfaction that can lead to enhanced overall wellbeing in the academic setting. Additionally, the mediating role of trust in leader is focused as a vital psychological element. While the results show a significantly positive effect on job satisfaction in the presence of empowering leaders, the moderating role of LMX alongside mediating impact of trust are observed. This implies that empowering leaders are highly influential in enhancing workplace for university teachers in the post-pandemic era.

## Introduction

The education sector was severely impacted by the COVID-19 pandemic as its traditional form was changed to online classrooms. In this regard, various scholars have reported that processes of learning, interactions, and cognitive and technological aspects were influenced for both teachers and students (Cachón-Zagalaz et al., 2020; De la Fuente et al., 2021; Telyani et al., 2021; Khawand and Zargar, 2022). Hence, the context of this study falls within the premises of educational psychology (within organizational psychology spectrum) that focuses on psychological factors in this sector specifically (De la Fuente et al., 2021). The pandemic forced teachers to work from home, by which several psychological issues arose, such as stress, anxiety, low engagement and motivation, work-life balance (Cachón-Zagalaz et al., 2020; Martarelli et al., 2021; Khawand and Zargar, 2022). While the aforementioned issues hindered psychological wellbeing of teachers during the pandemic, this study focuses on their job satisfaction in the post-pandemic era and under a positive leadership style (i.e., empowering). Importantly, this research also emphasizes on the environment, in which trust and interactions among leaders and their followers are enhanced to increase job satisfaction for academicians. Hence, this study takes job satisfaction as an important element that can determine teachers’ wellbeing after the pandemic and aid them with an appropriate workplace to thrive and develop both personally and professionally. Accordingly, this research argues that empowering leadership is an adequate approach in the academic setting that can yield in positive outcomes for teachers, considering the high demands of their jobs that are often combined with low amount of resources (Sarwar et al., 2021; Khawand and Zargar, 2022; Zaman et al., 2022).

Empowering leadership is regarded as a positive and ethical style that disregards the traditional flow of power by emphasizing on empowerment, and support, which lead to desirable outcomes for staff as well as the organization (i.e., university). This is due to the fact that such leaders can empower their followers from sociocultural (practices, interventions, and tactics for empowerment) and psychological aspects [self-determination, meaning, competence, and influence (Amundsen and Martinsen, 2015)]. Therefore, these leaders are found to be a good fit for the academic setting management (e.g., Amundsen and Martinsen, 2015; Farmanesh and Zargar, 2021; Jia et al., 2022). Within the context of this study, it is important to note that empowering leaders can boost trust (Farmanesh and Zargar, 2021) among their staff due to their behavior, and motivational approach that encourages positive exchange and interactions. This lies within the premises of leader-member exchange (LMX; Zhou et al., 2021), and its moderating impact on job satisfaction of university teachers is under examination in this research. Palestine, similar to other countries, had to face the challenges of the pandemic and its education sector shifted to online learning. However, in its current status, the universities are back to their normal settings. The current research aims to contribute to the extant literature of empowering leadership by examining its effects in academic sector with the inclusion of LMX, and trust in leader as determinants of job satisfaction. There is a vivid scarcity in the literature, when it comes to the Middle East and especially, Palestinian academic sector, which is regarded as a major driver for this study. In this sense, the core aim of this study is to investigate the relationship between empowering leadership and job satisfaction. The problem arises when in certain areas (i.e., Palestine) leadership style can differ from what is appropriate in the educational setting. Notably, the conduct of this research can be beneficial for scholars as it expands the applications of theories used in this study. Furthermore, practitioners in universities can benefit from the results of this research as it highlights the importance of adequate communication among leaders and followers in academic setting. Similarly, adequate leadership style in academic setting can foster trust-building, which is highly effective in determining the work outcomes (i.e., job satisfaction). Scholars have abundantly reported the positive impacts of empowering leaders on various employee and organizational outcomes including and not limited to, job satisfaction (Atik and Celik, 2020), trust (Farmanesh and Zargar, 2021), engagement (Helland et al., 2020), motivation (Muijs and Harris, 2003), commitment (Limon, 2022), and extra-role behaviors (e.g., organizational citizenship behavior; Bogler and Anit, 2004). Such effects are highly influential in shaping individuals’ careers in long-term as they provide the necessary atmosphere for growth and development (Zhou et al., 2021).

Following what was mentioned above, this study further justifies its conduct based on the male-dominant culture that persists in the leadership domain among Eastern cultures (Zhou et al., 2021) that can have different impacts on employees in the education sector, when compared to those in Western nations. Hence, this study has the potential to expand the geographical borders of existing knowledge on empowering leadership, and the applicability of LMX, job demand-resources model, and social exchange theories. As the current study highlights the gaps related to academic sector leadership, understanding empowering leaders’ effectiveness in university settings, and the context of Palestine, it is expected that findings can contribute to theoretical understanding in the extant literature as well as providing tangible means for practitioners (i.e., university managers) to better implement leadership styles that can improve the overall performance of their staff, enhance their wellbeing, and foster an environment, where individuals can trust their leaders, and thus, perform better in their roles. As the overarching outcome, current findings can pave the way for a better learning environment for students, as their teachers are enabled to work better, and are more satisfied with their working conditions (e.g., work environment, leadership approach, and communications). In accord with what was mentioned, the gap pertaining to Palestinian academic sector and its leadership styles is addressed in this study. This highlights the purpose of the research that endeavors to provide empirical evidence from this sector in Palestine as the literature shows scarcity.

The proposed model of this research falls within the educational psychology domain and it pertains to the aforementioned theories. In this sense, the issue that is tackled in this research is that academic sector of Palestine needs to be improved and encompass leaders that actively promote positive attitudes and behaviors that yield in organizational success for universities. Notably, the positive effects on university teachers’ wellbeing and performance will inevitably translate into an atmosphere, where students can thrive and benefit their societies in the future. Additionally, this study can be beneficial for understanding leadership styles that are effective in Palestine, and thus, provide empirical evidence that suggests tangible means for university decision-makers in this context. This can be used by managers in universities at the top level to recruit and/or develop leaders in their organizations that understand and value empowerment, while focusing on psychological wellbeing of their staff through building trust, and improving communications and interactions that can improve the work environment.

The role of leaders in academic setting is highly important as they can influence the overall outcomes of the university, teachers’ psychological wellbeing, and ultimately, the environment, in which students learn to build their futures. Hence, the current research emphasizes on the vitality of having satisfied teachers that are nurtured and empowered by their leaders through high-quality communications and interactions, and a focus on trust-building. The contextualization of this model is aimed at enhancing academic setting for teachers so that they can better perform their tasks and help shape the future of the country as they are more satisfied with their jobs. The problem is that not only there is scarcity in the literature regarding Middle East and Palestine, there is a need for better understanding leadership within academia as it affects the society (e.g., having satisfied teachers can benefit students as teachers are more likely to perform better). Considering the changes during and after the pandemic that occurred in the academic sector as well as the social, political, and economic issues of the country, the current research can pave the way for future studies addressing leadership in education sector, and psychological work outcomes (i.e., job satisfaction). To achieve the aforementioned aims, the following sections highlight the theoretical setting of the study while presenting its hypotheses. This is followed by detailed explanation of research methods and procedures (i.e., design, survey development and measures, data collection, ethical considerations, and deployed analytical technique). After this stage, the proposed model of the research is analyzed and results are discussed upon. Lastly, conclusions are noted alongside theoretical and practical implications as well as limitations that hindered this study in terms of conduct, and subsequent recommendations for future studies.

## Hypothesis development

### Theoretical setting

To shape the hypotheses of the study, and considering its aims and scope, a number of theories are used in this study that encompass relevant elements explaining the current context. These theories are: (a) Social exchange theory, which entails reciprocation as an important element for positive work outcomes (Mahmud et al., 2021; Haider et al., 2022). Within the workplace, staff are enabled to exchange knowledge and information in an adequate manner that is deployed by the leader. This has been noted as “responsible leadership” in the extant literature (see Haider et al., 2022). In this study, individuals in academic setting can have higher rate of job satisfaction as ethical and positive leaders act responsibly and therefore, significantly enhance the workplace for their staff through constructive exchanges. Sharing knowledge and expertise are key aspects of an exchange within the workplace that leaders can engage in, which subsequently, improves employees’ satisfaction. Within the context of academia, having satisfied employees is imperative as psychologically, they will be able to provide a better service (i.e., teaching) to students. (b) Job Demands-Resources model is also embedded in the current research as it encompasses both tangible and intangible aspects of work. Within the current research, it is argued that education sector is a highly demanding industry, where teachers and academic staff carry a number of responsibilities (Telyani et al., 2021; Khawand and Zargar, 2022). In addition to the aforementioned demands, the resources available for universities are often scarce and/or limited. Importantly, as the pandemic shifted the workplace of education organizations into online settings, the return to office after the pandemic is yet to be adequately examined. In terms of insufficient resources, various aspects can be noted, such as low wages, lack of long-term contracts, long hours of work, and additional tasks (e.g., exams, paperwork, research). As university teachers are required to use both mental and physical resources, and factors, such as anxiety, loneliness, work-life conflict, and performance have been hindered during the pandemic, it is argued whether empowering leaders can be a facilitator of change and enhance job satisfaction by establishing trust and appropriate practices within the organization (Atik and Celik, 2020; Helland et al., 2020; Tripathi et al., 2021; Haider et al., 2022). Considering the context of the current study, empowering leaders recognize the demands and resources, and strategically plan according to the limitations and capital that organization possesses.

In addition to what was noted, (c) Leader-Member exchange theory is also included in the current study as a managerial approach, in which leaders (i.e., empowering) take initiative in building and developing positive, engaging, and constructive exchanges in their relationship with employees, which establishes a nurturing environment, where information (e.g., knowledge) can be shared freely (Rehman et al., 2021). The importance of knowledge and its management within academic sector has been linked to gaining competitive advantage, developing organizational learning processes, and positive workplace outcomes. Notably, focusing on wellbeing of staff as well as providing a platform for their opinions and voices to be heard and/or implemented (where possible) can greatly improve the experience of individuals in the academic work setting (Martono et al., 2020; Rehman et al., 2021). The importance of exchanges and interactions between leaders and their followers is under examination in this research in relation to job satisfaction as a positive outcome. In this regard, the current study combines the premises of aforementioned theories to explain the current context and highlight its contributions. Following what was noted, through enhanced exchanges, leaders can create an environment where employees are encouraged to exhibit and support such behavior, which can have a positive impact on service quality that is provided to students (Yasmin et al., 2021). This states that the benefits of having satisfied employees in the academic sector can have deeper levels as it benefits the future generations in the learning environment.

### Empowering leadership and job satisfaction

Empowering leadership (hereafter EL) distinguishes itself from other leadership styles, such as transactional, transformational, and inclusive (Bolin, 1989; Amundsen and Martinsen, 2015; Capaldo et al., 2021). Empowering leaders tend to delegate tasks among their followers, include and involve them in decision-making processes, provide support, and increase job autonomy particularly in an academic setting, which can motivate teachers along other positive outcomes (i.e., job satisfaction; Wang and Yang, 2021). EL is noted to be comprised of competence, meaning, autonomy, and impact (Kim et al., 2018; Knezović and Musrati, 2018; Limon, 2022). In the context of university teachers and academic sector, EL manifests in a number of aspects that are namely, participation in decision-making processes (classroom management, and other school or departmental activities), professional development (opportunities perceived by teachers to grow and/or gain skills), status (perception of respect and appreciation), self-efficacy (teachers’ perception on their skillfulness and competence), autonomy (the extent of which teacher perceives control over their jobs), and impact (teachers’ perception of the level of their influence in the organization; Knezović and Musrati, 2018; Kim et al., 2018). Social exchange theory (SET; Blau, 1964) is embedded in this study as it addresses reciprocation behavior that occurs in the face of ethical, beneficial, and empowering behaviors of leaders toward university teachers (Helland et al., 2020; Wang et al., 2020). This theory also encompasses sharing knowledge and expertise within the workplace, which can greatly benefit employees and enhance the work environment (Rehman et al., 2021; Yasmin et al., 2021; Haider et al., 2022). When knowledge is shared and managed under a responsible leadership, who advocated ethics and empowerment for their staff, it is more likely that the experience of individuals is improved and thus, the likelihood of yielding in positive outcomes is increased.

Therefore, this study assumes that by enhancing work conditions and attributes, based on empowering leaders’ characteristics, job satisfaction of university teachers can be increased as they are more motivated and engaged (Amundsen and Martinsen, 2014). When teachers are provided with an atmosphere of prosperity, support, and development that includes autonomy, and encourages impact, it is more likely that their satisfaction level rises. Consequently, this can have positive impacts on psychological wellbeing and performance of teachers (Short and Rinehart, 1992; Wu and Short, 1996; Telyani et al., 2021; Khawand and Zargar, 2022). Importantly, in the current context, teachers in the university level are required to perform a number of tasks, while carrying the role of teacher. This can exhaust personal resources available to the individual especially when a crisis occurs, which can greatly change the settings in which work is routinely conducted (i.e., office). In this respect, the current study aims to contribute to the current understanding of job satisfaction among university-level teachers, which as a psychological aspect is highly influential in determining positive work and behavioral outcomes (Rehman et al., 2021; Haider et al., 2022).

Similarly, the JD-R model (Bakker and Demerouti, 2007) also fits in the current context as it addresses physical and psychological aspects of teachers’ job that is highly demanding and can lack in adequate resources (Khawand and Zargar, 2022) particularly, in the case of Palestine due to the general short-comings of the country (e.g., economic, social, and political issues; Aboramadan, 2020). This theory encompasses availability of resources (both personal and provided by the organization) to employees to perform their tasks. In this research, it is conceptualized that autonomy and competence can be promoted for teachers that can enhance their work processes and mental resources that they need (Atik and Celik, 2020; Helland et al., 2020). In contrast, when resources are scarce and demands are high (i.e., education sector and teaching profession), negative outcomes can arise such as decreased job satisfaction, lack of motivation and engagement, burnout, and work-life conflict (e.g., Aboramadan, 2020; Dahleez and Aboramadan, 2022; Khawand and Zargar, 2022). The role of appropriate leadership (e.g., empowering, responsible, and ethical) is highly influential in improving work environment for employees considering the limitations that the organization face (i.e., during and after the pandemic), and available resources (e.g., economic, risk management and policy and change management). Recent studies have emphasized on the importance of such matters in the academic setting (e.g., Helland et al., 2020; Rehman et al., 2021; Telyani et al., 2021; Yasmin et al., 2021; Haider et al., 2022), which further drives the conduct of current study, as additional empirical evidence is needed to develop the existing literature. Using the premises of JD-R model and SET, the current study emphasizes on leaders’ impact on overall improvement and development of academic workplace (i.e., university). Through empowering employees and particularly, teachers, the overall performance of the university can be improved as an overarching outcome linked to satisfied employees, who provide high-quality classes to their students.

As university teachers carry a number of responsibilities (e.g., teaching, exams, research, registration, and mentoring), provision of necessary tools and resources alongside organizational support and empowerment by their leaders become vital for their job satisfaction and performance outcomes that affect their wellbeing (Amundsen and Martinsen, 2015; Telyani et al., 2021; Zhou et al., 2021; Jia et al., 2022; Zaman et al., 2022). Job satisfaction of teachers is considered to be an emotional state toward work that entails subjective views, and attitudes toward the job itself (Zhou et al., 2021). Following what was mentioned, this research argues that empowering leaders can negate demands through delegation and increase resources *via* provision of support for teachers, which can positively affect job satisfaction. In addition, EL focuses on impact and meaning for teachers, which recognizes their value in the organization and enables involvement and influence in work processes. Based on the aforementioned arguments, the following hypothesis is shaped:

*Hypothesis 1*: EL has a positive and direct impact on job satisfaction of university teachers.

### Moderating role of LMX

The concept of LMX pertains to the relationship between leaders and their followers. This relationship is linked to the extent of which teachers, perceived interactions with leaders as close, honest, and high quality (Zhou et al., 2021). When employees perceive connectedness with their leaders, they are more likely to exhibit trust (Atik and Celik, 2020) and feel emotional attachments. This is embedded within the premises of SET as leaders can influence emotions of followers *via* positive, ethical, and effective communications that promotes reciprocation and trustworthiness (Farmanesh and Zargar, 2021; Zhou et al., 2021). Importantly, when employees have “good” exchanges with their leaders, they are prone to be more engaged and involved with their jobs, which can have positive outcomes (i.e., job satisfaction; Atik and Celik, 2020; Martono et al., 2020). Furthermore, to improve the academic work setting, having leaders, who engage in constructive, knowledge-based, valuable, and developmental (both personal and professional) exchanges with their followers is imperative. This is vivid in this context as considering university teachers’ knowledge, skills, and ability to manage classes requires a leadership, where opinions and voices are heard (Rehman et al., 2021; Haider et al., 2022), and their psychological and physiological wellbeing is cared for (Yasmin et al., 2021; Khawand and Zargar, 2022). Referring to the moderating role of LMX, the current study conceptualizes that through adequate leadership, the exchanges among leaders and teachers in university can create an atmosphere, where ideas and opinions are heard, while management endeavors to implement and optimize the workplace accordingly. Furthermore, sharing knowledge and expertise initiated by leaders can trigger reciprocity, where positive exchanges can occur across all levels and among employees following the behavior of the leader.

The current study examines the effect of social exchanges between an empowering leader and their followers in an academic setting that encompasses resources, and emotions of teachers being supported (Zhou et al., 2021). In this sense, contribution level, loyalty behavior, and respect (status) play an important role in determining the quality of exchanges between leaders and members. It is argued in this study that EL can implement high-quality interactions as it emphasizes on empowerment, support, and provision of necessary means (physical or mental support) for teachers in universities. As SET and JD-R models are linked to this context, empowering leaders can foster positivity, trust, and effectiveness in the workplace with a focus on employees’ wellbeing, which, in turn, can enhance their job satisfaction (Martono et al., 2020; Dahleez and Aboramadan, 2022).

**Figure 1:**
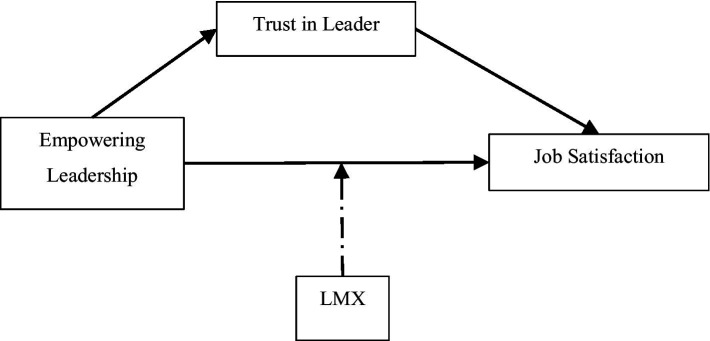
Research model.

- There are a limited number of studies that address this notion in the context of Palestinian education sector (e.g., Alkadash, 2020; Aboramadan et al., 2021). Notably, the proposed model of this research (see Figure 1) includes LMX as a moderating variable that enhances the relationship between empowering leaders and job satisfaction of their employees in university setting. In this regard, the importance of trust is also examined, which is explained in the following section. Empowering leaders enable participation for teachers in decision-making, recognize their skills and contributions to the organization, provide means for professional development, show respect to teachers, and implement their voice and ideas in work processes (Bruhn, 2006; Li and Zhang, 2016; Atik and Celik, 2020; Elkhwesky et al., 2022). Linked to the concept of LMX and SET, the behavior of empowering leaders creates bonds with their staff that are based on interactions, effective communication, and care. It is argued in this research that the aforementioned characteristics of EL are influential on job satisfaction of teachers in universities and can be further enhanced through appropriate and high-quality exchanges among followers and leaders. Accordingly, the following hypothesis is developed:

*Hypothesis 2*: LMX moderates the relationship between EL and job satisfaction.

### Mediating role of trust in leader

Trust in leader is regarded as a vital element in organizational setting as it encompasses an emotional state, where employees feel safe, cared for, and perceive ethical behavior in the conduct of their leaders (Farmanesh and Zargar, 2021). Empowering leaders can foster trust by creating an atmosphere, where teachers feel important, heard, valued, included, and supported (Farmanesh and Zargar, 2021). Employees can manage risk more effectively, show more involvement and creativity, and have more self-efficacy and job satisfaction, when the leaders empower sense of trust through their supportive behavior (McAllister, 1995; Zhang and Zhou, 2014; Javed et al., 2018; Martono et al., 2020). Trust in leader is noted to be in line with confidence, where both parties tend to maintain the trust by avoiding exploitations of vulnerabilities (Zargar et al., 2019). Leaders’ behavior is a key determinant in fostering trust within an organization (Liu et al., 2010). In this sense, the current research focuses on SET as the premise, in which ethical approach of leaders through EL will trigger trust and connectedness as it provides support and autonomy, and enables inclusion for teachers in university decision-making processes (Li et al., 2017; Lee et al., 2018). Empowering teachers psychologically can have a direct influence on their job satisfaction (Li et al., 2017; Zhu et al., 2019), leading to enhanced wellbeing and performance (Zhu et al., 2019; Razeq, 2022).

Employees feel secure and engage in pragmatic behaviors, when trust exists between them and their leaders (Atik and Celik, 2020). Persistence of trust has numerous constructive impacts on the psychology of teachers, which can lead to enhanced performance (Çelik and Konan, 2021; Tripathi et al., 2021), more engagement (Zhou et al., 2021), and significant rise in job satisfaction (Short, 1998; Gkorezis, 2016; Siachou et al., 2020). Therefore, it can be interpreted that the trust in leader can better explain the relationship between leader’s behavior (i.e., empowering), and employees’ psychological outcomes toward work (i.e., job satisfaction). As a vital psychological element, trust plays a major role in organizational settings and thus, its importance cannot be neglected (Mineo, 2014; Siachou et al., 2020; Farmanesh and Zargar, 2021). A leader who uses empowerment as the core approach and takes responsibility in action to provide care for mental wellbeing of their staff can exhibit behaviors that trigger trust especially within an academic setting (Rehman et al., 2021; Haider et al., 2022). This can translate into having empowering leaders within universities while conducting more examination on the subject. In the current context, trust can be nurtured by leaders’ who engage in interactions and conversations (LMX) that is directed toward benefiting the employee while sharing knowledge, policies, and ideas. This combined with overall positive behavior of the leader can trigger reciprocation (SET) through engaging in constructive and developmental exchanges that are focused on wellbeing (Yasmin et al., 2021; Haider et al., 2022). Based on the theoretical setting of the study and its aims and objectives, a hypothesis is merged:

*Hypothesis 3*: Trust in leader has a mediating effect on the relationship between EL and job satisfaction.

## Methodology

### Research approach and design

Based on the context of this research, a deductive quantitative approach is undertaken to test the hypotheses and achieve the set goals. Notably, many scholars have deployed a similar approach toward the subject as it addresses perception of employees (teachers in this case) regarding the behavior of their leaders (e.g., Amundsen and Martinsen, 2015; Atik and Celik, 2020; Helland et al., 2020; De la Fuente et al., 2021; Zhou et al., 2021; Limon, 2022). The hypotheses of the research are illustrated in Figure 1. A questionnaire was developed using available measures in the extant literature, and sample of questions are provided in the next section. Several universities were contacted by the first author using personal networks. It was established that empowering leadership is used in the selected universities to match the context of the study through a purposive approach. This inclusion criterion enables the researchers to ensure that participants are in fact interacting with empowering leaders. Several meetings (online and in-person) were held with department managers (i.e., deans), where information about EL was provided and upon confirmation, the university was selected for data collection. Relevant permissions were obtained from authorities in each university.

### Measurements

The self-administered questionnaire for this study is designed using relevant, valid, and available scales in the extant literature of the subject. In this respect, empowering leadership as the independent variable is measured through its dimensions (i.e., competence, meaning, autonomy, and impact) using a 10-item scale. These dimensions encompass leaders’ behavior and approach as well as psychological empowerment (Amundsen and Martinsen, 2015; e.g., my leader supports me in taking initiatives; my leader recognizes my contributions and skills; the work I do is meaningful; and I have influence in what happens in my department) (*α* > 0.7) (see Table 1). Trust in leader as a mediator was measured through organizational trust inventory (Nyhan and Herbert, 1997) using five items (e.g., I trust my leader because of his/her integrity) (*α* = 0.813). LMX as the moderator in the current model was measured through three questions (e.g., my leader is a person to befriend with; and I enjoy interactions and communications with my leader; Scandura and Graen, 1984; Zhou et al., 2021) (*α* = 0.724). Lastly, a short version of job satisfaction scale (Weiss, 2002) was used comprising five questions (e.g., my leaders’ behavior satisfies me; and I am satisfied with the organization I work with) (*α* = 0.765). This scale has been used to fit in the context of academia (Zhou et al., 2021). All questions are designed in a 5-item Likert scale ranging from 1 = totally disagree, to 5 = totally agree.

**Table 1:** Measurement model assessment.

Construct	Dimensions	Indicator	Outer loadings	α	Rho A	CR	AVE	VIF	Weights	*t*-stat.	CV
Empowering leadership	*Competence*	CP1	0.711	0.807	0.807	0.785	0.613	2.014	0.409	2.341**	0.719
		CP2	0.802						0.422	2.211**	
	*Meaning*	MN3	0.845	0.784	0.813	0.761	0.707	1.889	0.361	2.188*	0.701
		MN4	0.807						0.470	4.004**	
	*Autonomy*	AU5	0.823	0.731	0.776	0.719	0.631	2.120	0.413	3.647**	0.713
		AU6	0.757						0.324	2.578*	
	*Impact*	IM7	0.883	0.724	0.744	0.726	0.723	2.244	0.355	2.649**	0.708
		IM8	0.862						0.386	2.178*	
		IM10	0.853						0.502	3.805*	
Trust in leader	–	TIL1	0.803	0.813	0.870	0.762	0.739	–			
		TIL2	0.734								
		TIL3	0.728								
		TIL4	0.798								
		TIL5	0.804								
LMX	–	LMX1	0.720	0.724	0.779	0.722	0.656	–			
		LMX2	0.753								
		LMX3	0.761								
Job satisfaction	–	JS1	0.832	0.765	0.811	0.873	0.616	–			
		JS2	0.860								
		JS3	0.831								
		JS4	0.876								
		JS5	0.855								

*0.05; **0.01; ***0.001.

### Sampling procedure

Using G*power software (Faul et al., 2007), and recommendations of experts (Hair et al., 2017), the sample size was calculated at 163 with a specific criterion that meets satisfactory statistical prowess (statistical power = 85%, Min *R*^2^ = 0.10, and *α* = 0.01). Hence, the desired sample size should not fall below 163. A pilot test was deployed with 20 participants, where readability and validity of items were tested and found significant. Upon completion of pilot test, a total of 250 questionnaires were distributed among teachers of 6 universities across Palestine based on their willingness to participate and availability. The survey was translated into Arabic by a professional and translated back into English using a second translator to ensure accuracy of terms (Podsakoff et al., 2012). Surveys were shared *via* email during July 2022 and respondents were given 3 days to return their responses. Convenience sampling technique was used for gathering data from participants. To ensure ethical means of conduct, several aspects were taken into consideration, namely each participant was informed of research purposes and objectives; written consent form was provided to participants; participation was voluntary and withdrawal was made possible at any stage; no personal information was collected and confidentiality was given to participants; and original data were deleted upon computerization of responses. These measures ensured compliance with ethics while reducing the rate of common method bias (Podsakoff et al., 2012). A total of 180 surveys were returned, from which 2 were incomplete and thus, were not qualified for final analysis, leaving 178 responses for data analysis (71.2% response rate). Collinearity test was deployed, where variance inflation factor (VIF) values were below the threshold of 3, implying that common method bias (CMB) is not a concern (Kock, 2015; see Table 1).

### Control variables

Demographic factors included in the survey (i.e., age, gender, and years of experience) were controlled in the analysis based on their impact on perception toward leader, job satisfaction, and trust. These measures were undertaken based on the extant literature and relevant studies in the same context (e.g., Amundsen and Martinsen, 2014; Helland et al., 2020; Zhou et al., 2021).

### Respondents’ characteristics

As noted above, to reduce CMB and ensure confidentiality, personal/demographic variables included in the survey were age, gender, and years of experience. The results in Table 2 show that the number of women (*n* = 91) and men (*n* = 87) do not differ significantly. Moreover, majority of participants were between 30 and 35 years of age (38%) followed by 36–41 (32%), 42–47 (19%) and above 47 (11%). Notably, majority of participants had above 5 years of experience (61%), which implies their understanding, awareness, and level of know-how in the academic setting.

**Table 2:** Demographics.

Factor	Item	Frequency
Age	30–35	68
	36–41	57
	42–47	34
	above 47	19
Gender	Male	87
	Female	91
Experience (years) *N* = 178	Less than 5	49
	5–10	67
	More than 10	62

### Analysis and results

Smart-PLS software was used to analyze the obtained data through a specific criterion that fits the deployed analytical technique of this research (i.e., Partial Least Squares–Structural Equation Modeling; PLS-SEM). This technique is deemed appropriate for the current study as it entails latent variable, small sample size that requires statistical power, and does not concern normality of distribution in the data (Lowry and James, 2014; Hair et al., 2019). As the current study aims to investigate empowering leadership-job satisfaction relationship empirically, it is deemed appropriate that quantitative method is applied. Furthermore, to adequately analyze moderation and mediation effects of LMX and trust in leader respectively, PLS-SEM is an analytical technique that can appropriately test the hypotheses and yield in tangible results (considering the specific criteria and framework of the study).

### Measurement model assessment

The results presented in Table 1 suggest that the measurement model is acceptable as reliability and validity values are within the satisfactory thresholds. In this respect, internal consistency (Rho A and α) and composite reliability (CR) are found to be above 0.7 and below 0.9, stating satisfactory values for these measures (Kroonenberg et al., 2006; Diamantopoulos et al., 2012; Dijkstra and Henseler, 2015). Additionally, outer loading are found statistically acceptance as they are above 0.708 (Hair et al., 2019). Moreover, Table 1 shows that average variance extracted (AVE) is above 0.5. This implies that convergent validity (CV; Dijkstra and Henseler, 2015) is adequate for the proposed model (Hair et al., 2017).

Table 3 shows the result of heterotrait-monotrait (HTMT), which states that discriminant validity of measurement model is adequate as it does not surpass 0.85 (Henseler et al., 2015). Combined results of Tables 1, 3 provide a satisfactory level for the measurement model, which implies it appropriateness and adequacy.

**Table 3:** Heterotrait-monotrait (HTMT).

	EL	CP	MN	AU	IM	TIL	LMX
EL							
CP	0.765						
MN	0.566	0.523					
AU	0.637	0.613	0.770				
EIM	0.619	0.707	0.684	0.722			
TIL	0.693	0.580	0.671	0.744	0.820		
LMX	0.744	0.701	0.626	0.751	0.775	0.811	
JS	0.623	0.657	0.658	0.667	0.721	0.703	0.731

### Structural model assessment

Table 4 presents the results of structural model assessment (hypothesis testing). The model meets the requirements, namely (1) normal fit index (NFI = 0.920) and standardized root mean square residual (SRMR = 0.023) are representations of a “good model fit” (Henseler et al., 2014); (2) no multicollinearity issues were noted (VIF < 3; see Table 1; Hair et al., 2019); both in-sample predictive power (R-square) and predictive relevance (Q-square) are within acceptable range of values (Henseler et al., 2009), as shown in Table 4. As it can be observed from Table 4, the direct and significantly positive impact of EL on job satisfaction is proven (*β* = 0.307), which provides support for the first hypothesis of the research. Similarly, the moderation effect of LMX on the EL-job satisfaction relationship is found to be statistically significant (*β* = 0.136). This supports the second hypothesis of the research. Lastly, mediation effect of trust in leader is also found to be significant (*β* = 0.144), which leads to acceptance of the third hypothesis of the research.

**Table 4:** Hypothesis testing.

Effects	Relations	*β*	*t*-statistics	Ƒ^2^	Decision
Direct					
H1	EL → JS	0.307	5.227***	0.122	Supported
Interaction					
H2	EL*LMX → JS	0.136	2.846**	0.041	Supported
Mediation					
H3	EL → TIL → JS	0.144	2.979**	0.033	Supported
Control variables					
	Gender → JS	0.151	2.578*		
	Age → JS	0.132	2.655*		
	Experience → JS	0.166	2.702*		
R^2^_JS_ = 0.49 / Q^2^_JS_ = 0.29 R^2^_TIL_ = 0.67 / Q^2^_TIL_ = 0.47 SRMR: 0.023; NFI: 0.920					

*0.05; **0.01; ***0.001.

## Discussion

As this research focuses on the benefits of empowering leadership in an academic setting in the Middle East (i.e., Palestine), job satisfaction was analyzed as a representation of psychological wellbeing within the context of educational psychology. In addition, the moderating effect of LMX as an enhancer was analyzed in this context with a focus on the quality of interaction between empowering leaders and their followers (i.e., university teachers). Furthermore, trust in leader as a critical psychological factor was examined based on its mediating effect on the relationship between EL and job satisfaction of university teachers.

Vivid positive effects of EL were noted in the results, which shows consensus with prior studies in this context (e.g., Atik and Celik, 2020; Farmanesh and Zargar, 2021; Zhou et al., 2021; Jia et al., 2022). It was also noted in the literature that positive attributes of leaders can lead to notable positive attitudes toward the job and the organization (Amundsen and Martinsen, 2015). In the same context, studies have also reported that empowering leadership is a determinant of creativity due to delegation and autonomy that is provided to employees (Short and Rinehart, 1992; Wu and Short, 1996; Atik and Celik, 2020; Dahleez and Aboramadan, 2022). This study argues that by increasing autonomy for university teachers, they are more likely to be engaged and involved with their jobs, and use new approaches or techniques to better perform their tasks. When leaders empower teachers in the university setting, job satisfaction will increase and thus, performance can be enhanced. This can further be achieved by provision of professional development programs to increase competence level of teachers. The current findings while being in line with previous findings add to the current understanding of EL in the context of Middle East and particularly, Palestine, which is understudied in terms of leadership, and its academic sector. These results are embedded within the premises of SET and JD-R (Alkadash, 2020; Helland et al., 2020; Limon, 2022) as they describe the relationship between leader and follower in academic setting that is a high-demanding sector, which in Palestine specifically, is often parallel with lack of adequate resources (Alkadash, 2020; Aboramadan et al., 2021).

Pertaining to second hypothesis of this study, moderating role of LMX was found to be significant. Inclusion of LMX (high-quality interactions and exchanges between leader and teacher) enhances the effect of EL on job satisfaction. The positive impacts of LMX have been reported in the extant literature related to leadership, organizational outcomes, and psychological wellbeing (Khawand and Zargar, 2022). Based on SET (Blau, 1964) and LMX (Gottfredson et al., 2020) theories, leaders in universities can explicitly increase job satisfaction by focusing on effective communication and informing teachers on work settings, managerial decisions, and other processes. This also is linked to empowerment of teachers by promoting participation, provision of professional development opportunities, exhibition of respect, increasing autonomy, and implementing teachers’ ideas (Kim et al., 2018; Knezović and Musrati, 2018). These results are beneficial for understanding the importance of adequate leadership in academia. Empowering leaders are found to be a good fit in this context as they can foster necessary means to motivate, empower, and meet the needs of teachers in their demanding jobs.

Referring to the third hypothesis of this research, which was supported in the data analysis (see Table 4), mediating role of trust in leader was found statistically significant in the proposed model of this research. It is important to note that the vitality of trust in leader has been noted across the literature by a considerable number of scholars (e.g., Zhang and Zhou, 2014; Javed et al., 2018; Saleem et al., 2020; Farmanesh and Zargar, 2021; Faulks et al., 2021), stating that trust carries a major role in determining satisfaction, motivation, involvement, and other positive behaviors, such as creativity and commitment (Tsang et al., 2022). As trust is tied to complex psychological processes for each individual, behavior of leader is essential in fostering an environment, where employees can perceive trustworthiness, and ethical conduct. Through trust, teachers are more likely to have higher engagement with their work, which, in turn, will enhance the classroom environment for students (Telyani et al., 2021; Khawand and Zargar, 2022; Sun et al., 2022). Therefore, it can be interpreted from current results that teachers should be empowered by their leaders in a manner that establishes trust and encourages honest, clear, and effective communications. Linked to SET, teachers will be more willing to contribute to their organizations, when they feel that they can trust their supervisors/leaders.

It is also important to note that control variables of the research (i.e., age, gender, and experience) were found to be influential in determining job satisfaction of teachers in universities. However, as these effects were controlled, further research is required to shed light upon this matter. Based on what was mentioned above, the current results contribute to both theoretical and practical domains surrounding leadership (i.e., empowering), trust in leader, and job satisfaction literature. These implications can be beneficial for scholars and managers in the Middle East and specifically Palestine. Conclusions and implications derived from current findings are presented in the following sections.

## Conclusion

The results that support the conclusions and discussions of this study show that empowering leaders can explicitly improve job satisfaction of employees in an academic setting. Due to high extent of competitiveness in this sector (Muijs and Harris, 2003; Bouwmeester et al., 2021; Mayya et al., 2021), leaders’ role become more vital in maintaining an environment, where teachers can develop their professional skills while being satisfied of work settings and communications and interactions with their supervisors. With a focus on LMX and SET, empowered teachers are more likely to have their job satisfaction improved. Furthermore, characteristics of an empowering leader can manifest in an atmosphere of trust, where teachers are encouraged to show more engagement, involvement, and commitment. Therefore, the conclusions of this research can be summarized into (a) empowering leadership is an appropriate style in academic setting as it can increase job satisfaction; (b) empowering leaders can boost interactions with university teachers through LMX; and (c) by emphasizing on trust-building, job satisfaction of teachers can be better explained in the university level. The overarching outcome is improved psychological wellbeing of teachers in a job that demands their mental and physical resources. Moreover, it can be interpreted that through applying adequate leadership styles in the academic sector, students can benefit as the ultimate results. This is due to the fat that job satisfaction is a psychological factor that is a vital element for psychological wellbeing. Therefore, in the high-demanding sector of education, having satisfied teachers can greatly benefit the society by better preparing the next generation through engaging, positive, and innovative classes. Scholars can benefit from current results as the analyzed model of this study combines SET, JD-R, and LMX in terms of their premises. Notably, the enhancing effect of LMX can be beneficial for practitioners as emphasizing on developing and optimizing exchanges in the workplace can significantly improve job satisfaction of employees in university level. Furthermore, combination of the aforementioned theories can be used by scholars in different settings and using various techniques to better understand the impact of empowerment in the education sector of neighboring countries. This can further increase the benefits of current results as comparative evidence can yield in improved comprehension of the subject of leadership in academia.

### Theoretical implications

The current findings showed a positive impact on job satisfaction in the presence of empowering leaders in university setting. This is linked to the context of educational psychology as well as organizational psychology as broader concepts. Importantly, SET and JD-R (Blau, 1964; Bakker and Demerouti, 2007; Wang et al., 2020; Zhou et al., 2021) models are used in this research that address interactions and reciprocations in workplace, and physical and psychological resources needed to conduct the job of a university teacher. The findings show that empowering leaders can improve psychological wellbeing of university teachers in Palestine, implying the application and appropriateness of the aforementioned theories. While SET explains how EL can generate reciprocity in a positive way that can improve performance and engagement while contributing to job satisfaction as psychological wellbeing, JD-R pertains to the importance of providing necessary means for teachers to conduct their tasks. In this respect, this study suggests that empowering leaders recognize demands and resources of university teachers’ jobs and tend to provide development opportunities and mental and professional support, and implement different techniques (e.g., human resource management initiatives) that increase competence and skill level of these individuals. As respect, and recognition are important in the Middle Eastern culture (Behery et al., 2018), current findings imply that EL through SET can be highly effective for improving academic sector by empowering teachers.

Furthermore, this study embeds the premises of LMX theory (Scandura and Graen, 1984; Zhou et al., 2021) in the current context, which pertains to the extent of which leaders engage in high-quality interactions and communications with their staff. In this respect, the findings show that although LMX is not rooted among the characteristics of EL, its inclusion can lead to higher rates of positive outcomes (i.e., job satisfaction). While some individuals can have better relationships with their leaders (Zhou et al., 2021), current findings suggest that through LMX, an empowering leader can improve job satisfaction of teachers by focusing on enhanced interactions and exchanges. The concept of this theory can also be linked to the notion of trust-building as it pertains to honest, direct, and support that can yield in positive outcomes (i.e., psychological wellbeing; Rasool et al., 2019, 2022; Zhou et al., 2020; Zaman et al., 2022). Implementation of such approaches in universities as leadership strategies of the organization can overcome the negative impacts of the pandemic (e.g., stress, anxiety, and burnout; Telyani et al., 2021), while increase resilience for the university, when facing future crises (Ojo et al., 2021).

### Practical implications

First, shareholders in universities of Palestine (and Middle East region by extension) should recognize the effectiveness and adequacy of empowering leaders in education sector. By doing so, recruitment, attraction, and development of such leaders becomes a strategic plan, through which empowerment of employees at all levels turns into organizational mission. As these leaders consider different aspects of the job carried by their followers, they can improve the quality of workplace for teachers, which, in turn, can improve the learning process of their students. Secondly, deans and leaders in departments of universities should engage in healthy, positive, and honest communications with teachers and inform them of organizational decisions and processes. This will improve inclusion, involvement, and thus, job satisfaction. Thirdly, exhibition of respect and recognition of teachers’ contribution is highly important as it can create a positive attitude toward the leader and organization and develop trust. Fourthly, collaboration with HR departments in universities can lead to establishing support systems, counseling, and professional development programs that can improve teachers’ skills, wellbeing, and satisfaction. By implementing such initiatives, universities can have systems that not only benefit teachers during their work, but further prepare the organization for facing future crises as the implemented HR systems can play a major role in promoting work-life balance, and providing support both mentally and professionally (e.g., facilitating work conditions). Lastly, department deans (i.e., leaders) should hear the voice and ideas of their teachers and implement these ideas in the flow of organization. Where implementation of all ideas is not feasible, teachers should be provided with autonomy to conduct their tasks and manage their roles. Such initiatives can have both short- and long-term outcomes that benefit teachers, university as the organization, and subsequently, improve students’ learning environment.

### Limitations and recommendations

Regardless of findings and achieving research objectives, there are a number of con-straining factors that hindered the process of this study. In this regard, due to complexity of human behavior, there are other theories (e.g., social learning, achievement goal theory, signaling theory, self-determination, and social cognitive theories) that are linked to the context of this research. However, the context of this study was limited to SET, JD-R and LMX theories, which provided sufficient support for its conduct. Future studies can address this limitation and combine relevant theories to provide a better understanding on the subject at hand. In addition, this study was limited by the scarcity of studies that address the Middle East and specifically, Palestine. Therefore, we suggest that scholars provide more empirical evidence from this region to yield in more knowledge and awareness regarding leadership and education sector of the Middle East. Moreover, the data collection process was conducted in a cross-sectional manner, which does not include temporal links among variables. Future studies can deploy longitudinal technique to avoid this limit and examine changes in behavior of teachers in a period of time. Similarly, quantitative nature of this research is limited in terms of generalizability, and lacks in-depth understanding of the phenomenon at hand. This can be avoided in future studies that take a qualitative approach. Last but not least, cultural elements were not examined in the current study as they fall beyond its scope. Hence, future studies can analyze how cultural and social elements can impact teachers and leadership in education sector of Palestine.

## Data availability statement

The raw data supporting the conclusions of this article can be made available upon request from the corresponding author.

## Ethics statement

Ethical review and approval was not required for the study on human participants in accordance with the local legislation and institutional requirements. The patients/participants provided their written informed consent to participate in this study.

## Author contributions

IH: initial writing, model development, and data collection. PZ: data analysis, revision, review, software, and methodology. All authors contributed to the article and approved the submitted version.

## Conflict of interest

The authors declare that the research was conducted in the absence of any commercial or financial relationships that could be construed as a potential conflict of interest.

## Publisher’s note

All claims expressed in this article are solely those of the authors and do not necessarily represent those of their affiliated organizations, or those of the publisher, the editors and the reviewers. Any product that may be evaluated in this article, or claim that may be made by its manufacturer, is not guaranteed or endorsed by the publisher.
